# Lessons learnt from assessing and improving accuracy and positive predictive value of the national HIV testing algorithm in Nigeria

**DOI:** 10.4102/ajlm.v13i1.2339

**Published:** 2024-08-28

**Authors:** Augustine O. Mpamugo, Nnaemeka C. Iriemenam, Adebobola Bashorun, Olumide O. Okunoye, Orji O. Bassey, Edewede Onokevbagbe, Tapdiyel Jelpe, Matthias A. Alagi, Chidozie Meribe, Rose E. Aguolu, Charles E. Nzelu, Segun Bello, Babatunde Ezra, Christine A. Obioha, Baffa S. Ibrahim, Oluwasanmi Adedokun, Akudo Ikpeazu, Chikwe Ihekweazu, Talishiea Croxton, Sylvia B. Adebajo, McPaul I.J. Okoye, Alash’le Abimiku

**Affiliations:** 1 Center for International Health, Education and Biosecurity, Maryland Global Initiatives Corporation, University of Maryland, Baltimore (UMB), Abuja, Nigeria; 2 Division of Global HIV and TB, United States Centers for Disease Control and Prevention, Abuja, Nigeria; 3 National AIDS, Viral Hepatitis, and STIs Control Programme, Federal Ministry of Health, Abuja, Nigeria; 4 Department of Research Monitoring and Evaluation, National Agency for the Control of AIDS, Abuja, Nigeria; 5 Department of Planning, Research and Statistics, Federal Ministry of Health, Abuja, Nigeria; 6 Department of Epidemiology and Medical Statistics, Faculty of Public Health, College of Medicine, University of Ibadan, Ibadan, Nigeria; 7 Nigeria Centers for Disease Control, Abuja, Nigeria; 8 Institute of Human Virology, University of Maryland School of Medicine, Baltimore, Maryland, United States

**Keywords:** lessons learnt, HIV/AIDS, HIV rapid test, testing algorithm, Nigeria

## Abstract

**Background:**

HIV testing remains an entry point into HIV care and treatment services. In 2007, Nigeria adopted and implemented a two-test rapid HIV testing algorithm of three HIV rapid test kits, following the sequence: Alere Determine (first test), Unigold^TM^ (second test), and STAT-PAK^®^ as the tie-breaker. Sub-analysis of the 2018 Nigeria HIV/AIDS Indicator and Impact Survey data showed significant discordance between the first and second tests, necessitating an evaluation of the algorithm. This manuscript highlights lessons learnt from that evaluation.

**Intervention:**

A two-phased evaluation method was employed, including abstraction and analysis of retrospective HIV testing data from January 2017 to December 2019 from 24 selected sites supported by the United States President’s Emergency Plan for AIDS Relief programme. A prospective evaluation of HIV testing was done among 2895 consecutively enrolled and consented adults, aged 15–64 years, accessing HIV testing services from three selected sites per state across the six geopolitical zones of Nigeria between July 2020 and September 2020. The prospective evaluation was performed both in the field and at the National Reference Laboratory under controlled laboratory conditions. Stakeholder engagements, strategic selection and training of study personnel, and integrated supportive supervision were employed to assure the quality of evaluation procedures and outcomes.

**Lessons learnt:**

The algorithm showed higher sensitivity and specificity in the National Reference Laboratory compared with the field. The approaches to quality assurance were integral to the high-quality study outcomes.

**Recommendations:**

We recommend comparison of testing algorithms under evaluation against a gold standard.

**What this study adds:**

This study provides context-specific considerations in using World Health Organization recommendations to evaluate the Nigerian national HIV rapid testing algorithm.

## Background

HIV testing services (HTS) remain an entry point into HIV care and treatment.^1^ HIV testing is the first pillar of the Joint United Nations Program on HIV/AIDS (UNAIDS) 95-95-95 cascade,^2^ which helps people living with HIV to access HIV prevention and treatment services. In addition, HIV testing supports programmatic efforts to reach the UNAIDS target of 95% of people living with HIV becoming aware of their HIV status by 2030.^3^ As of 2020, Nigeria had about 1.8 million people living with HIV, representing about 5% of the global burden and 7% of the African burden by the end of 2020.^4^ With the support of donors and implementing partners, in 2020, the government of Nigeria made substantial progress towards attaining the UNAIDS 95-95-95 targets needed to achieve HIV epidemic control by 2030. Thus, by the end of 2020 in Nigeria, 73% of people living with HIV were diagnosed, 89% of those diagnosed accessed HIV treatment, and 78% of those who accessed HIV treatment were virally suppressed.^4^

The adoption of the Test and Treat Strategy^5^ for anti-retroviral therapy initiation underscores the importance of accurately diagnosing true positives. False positivity due to misdiagnosis will result in erroneously initiating such wrongfully diagnosed persons on lifelong anti-retroviral therapy, which would have significant emotional, financial, and psychosocial implications for the affected individuals.^6,7,8^ This places enormous responsibility on the country’s government to identify, adopt, and implement an appropriate HIV testing algorithm that complies with World Health Organization guidelines for HIV algorithm selection.^9^

Validating the national HIV testing algorithm is crucial to determining its optimal performance across testing streams.^10^ Before 2005, there was no formal evaluation of HIV rapid test kits for developing a national algorithm in Nigeria.^11^ In 2006, in collaboration with the United States President’s Emergency Plan for AIDS Relief (PEPFAR) programme, Nigeria implemented and completed two phases of evaluation; the first was a laboratory-based evaluation that rated nine HIV rapid test kits with a specific focus on non-cold chain-dependent rapid test kits to suit both infrastructure and the varied national skilled level.^11^ The second was a field evaluation of the interim algorithm to assess the test performance in a non-controlled environment and interpretations of the test by non-laboratory personnel such as HIV counsellors. The World Health Organization had recommended using rapid HIV tests, since the conventional enzyme immunoassay and Western Blot constituted a challenge and reduced access to testing, especially in resource-limited nations.^9^ Thus, the phased evaluations identified suitable algorithms based on combining two or more rapid tests with a diagnostic accuracy comparable to enzyme immunoassay and Western Blot strategies.^11^ Based on the initial evaluation report in Nigeria, three serial HIV testing algorithms were recommended for use in three combinations: (1) Determine^TM^ HIV 1/2 (Abbott Molecular Inc., Des Plaines, Illinois, United States), STAT-PAK^®^ HIV 1/2 Assay (Chembio Diagnostic Systems Inc., Medford, New York, United States) and Bundi^TM^ (Bundi International Diagnostics Ltd, Aba, Abia State, Nigeria); (2) Unigold^TM^ (Trinity Biotech, Plc., Wicklow, Ireland), STAT-PAK^®^ (Chembio Diagnostic Systems, Inc. Medford, New York, United States) and Bundi^TM^; and (3) Determine^TM^, Unigold^TM^ and STAT-PAK^®^.^11^ Consequently, Nigeria adopted a serial algorithm in 2007, which was a two-test algorithm comprising of Alere Determine HIV rapid test as the first test, Unigold^TM^ HIV rapid test as the second test, and STAT-PAK^®^ HIV rapid test as the tie-breaker.^11,12^

The two-test national HIV rapid testing algorithm was used during the 2018 Nigeria AIDS Indicator and Impact Survey to estimate the national HIV prevalence and incidence. The success of this survey gave a more accurate status of the national HIV prevalence in Nigeria compared to previous surveys.^10,13,14^ However, a sub-analysis of 2018 Nigeria AIDS Indicator and Impact Survey data revealed a concordance rate of 56.6% between the first and second tests for HIV tests performed in the field, indicating that the Nigeria HIV rapid testing algorithm may be performing suboptimally in these settings.^10^

This article presents lessons learnt from evaluating the performance of the national HIV testing algorithm in Nigeria using a two-phase approach that (1) retrospectively analysed HIV testing programme data collected from January 2017 to December 2019 from routine HIV testing sites across the six geopolitical zones of Nigeria, and (2) prospectively analysed collected samples from six geopolitical zones of Nigeria.

## Description of the intervention

### Ethical considerations

Ethical clearance for this evaluation was obtained from the National Health Research Ethics Committee of Nigeria (approval number 01/01/2007-22/05/20208) and the University of Maryland Baltimore Institutional Review Board (approval number HM-HP-00091258-1). This project was reviewed in accordance with United States Centers for Disease Control and Prevention (CDC) (Project ID: 0900f3eb81b4a2a1, Accession number CGC-NGRA-5/14/20-4a2a1) human research protection procedures and was determined to be research, but United States CDC investigators did not interact with human subjects or have access to identifiable data or specimens for research purposes.

### Setting

Nigeria has an estimated population of 220 544 164, based on the United Nations World Population Prospects (2022 Revision) estimate, and covers a land mass of 923 768 square kilometers.^15^ Nigeria has 36 states, and a Federal Capital Territory grouped into six geopolitical zones.

### Study design

A cross-sectional design was implemented. The evaluation was conducted in two phases: a prospective and a retrospective study.

#### Phase 1: Retrospective evaluation

The performance of the national HIV rapid testing algorithm was evaluated retrospectively by reviewing and analysing HIV testing programme data of de-identified patients from 24 purposively selected PEPFAR-supported sites in 12 states across the six geopolitical zones of Nigeria. Data were abstracted from the paper-based National Daily HIV Testing Register by trained data clerks. The criteria for site selection included HIV prevalence levels of the states and HIV testing volume of at least 1000 tests per year (Table 1). The period covered was January 2017 to December 2019. The abstracted data were entered directly into a custom-built electronic data collection platform.

**TABLE 1:** **States selected for the retrospective phase of evaluation of HIV rapid testing algorithm in** Nigeria, July 2020 – September 2020.

Selected states	HIV prevalence (%)	Prevalence level	Retrospective evaluation sites	Geopolitical zones
Katsina	0.3	Low	2	North West
Kaduna	1.0	Low	2	North West
Ekiti	0.8	Low	2	South West
Ogun	1.4	Medium	2	South West
Gombe	1.3	Medium	2	North East
Taraba	2.6	Medium	2	North East
Enugu	2.0	Medium	2	South East
Ebonyi	0.8	Low	2	South East
Rivers	3.8	High	2	South South
Delta	1.7	Medium	2	South South
Benue	5.3	High	2	North Central
FCT	1.4	Medium	2	North Central

*Source*: Adapted From: Iriemenam NC, Mpamugo A, Ikpeazu A, et al. Evaluation of the Nigeria national HIV rapid testing algorithm. PLOS Glob Public Heal. 2022;2(11):e0001077. https://doi.org/10.1371/journal.pgph.0001077

#### Phase 2: Prospective evaluation

**Sampling – State and site selection:** Sampling for this evaluation was done in six states across the six geopolitical zones, consisting of one state per zone. The six states were purposively selected based on their prevalence levels (low, medium, and high) according to the 2018 Nigeria AIDS Indicator and Impact Survey report,^16^ as well as security and safety considerations. Three HTS sites per state per zone were selected based on-site selection criteria: availability of HTS, low, medium, and high positive case yields, ease of transportation and shipments to the National Reference Laboratory (NRL), and security and safety consideration. In total, 18 HTS sites were used for the evaluation (Table 2).

**TABLE 2:** Purposively selected states for the prospective phase of the evaluation of HIV rapid testing algorithm in Nigeria, July 2020 – September 2020.

Selected states	HIV prevalence (%)†	Prevalence level	Sites for prospective evaluation	Geopolitical zones
Katsina	0.3	Low	3	North West
Ekiti	0.7	Low	3	South West
Gombe	1.2	Medium	3	North East
Enugu	1.8	Medium	3	South East
Rivers	3.6	High	3	South South
Benue	4.8	High	3	North Central

*Source*: Adapted From: Iriemenam NC, Mpamugo A, Ikpeazu A, et al. Evaluation of the Nigeria national HIV rapid testing algorithm. PLOS Glob Public Heal. 2022;2(11):e0001077. https://doi.org/10.1371/journal.pgph.0001077

Note: Low prevalence = 0.1–1.0; medium prevalence = 1.1–3.0; high prevalence ≥3.0.

† , HIV prevalence calculated using the Nigeria AIDS Indicator and Impact Survey prevalence level, which is based on the Nigeria AIDS Indicator and Impact Survey report of 2018 and not on the World Health Organization rating.

**Sample size:** The estimated sample size was 3000 (1800 negatives and 1200 positives), comprising 500 samples (300 negatives and 200 positives) per geopolitical zone. The actual sample collected was 2895, comprising 1817 negatives and 1078 positives, from participants aged 15 years – 64 years, who consented to and accessed HTS services from July to September 2020. A 10 mL ethylenediaminetetraacetic acid vacutainer was used to collect blood samples from all participants.

**Survey procedures:** Consenting survey participants were recruited, and capillary blood was drawn by finger sticks to perform the first test in the algorithm. When the first test was non-reactive, the participant was given a negative HIV result and escorted to the site’s laboratory for a venous whole blood collection into a 10 mL ethylenediaminetetraacetic acid tube. The same process was done for all HIV-negative results across the three sites until the targeted 300 HIV-negative samples were achieved for that state/geopolitical region. Subsequent negative results from the consenting participants were excluded from the study. Similarly, when the first test was reactive, the participant was also taken to the site’s laboratory, where whole venous blood was collected into a 10 mL ethylenediaminetetraacetic acid tube, and part of this sample was used to perform the second test. If the second was also reactive, the participant was confirmed HIV positive according to the national HIV testing algorithm. However, if the result of the second test was non-reactive, then a tie-breaker test was performed, and the result of the tie-breaker was taken as the final HIV test status of the participant. This process was conducted across the study sites for all participants who were reactive at the first test until the targeted 200 HIV positives per state/geopolitical zone were achieved. The remnants of the 10 mL blood samples were then separated by centrifugation (1500 × g for 10 min or ~3000 rpm) into plasma suspension, and the plasma was aliquoted into two cryo-tubes of about 1.5 mL each, and labelled with a pre-printed sticker containing de-identified patient identity codes comprising state, site, and alphanumeric codes. The codes were pre-printed as barcode labels to avoid documentation errors and improve tracking of sample shipments from the field to the NRL. The aliquots were packaged in cryo-boxes and shipped to the NRL in Abuja in −20 °C Credo boxes.

All plasma samples received at NRL were stored at −86 °C ultra-low freezers, Dw-HL 678S Zhongke Meiling (Zhongke Meiling Cryogenic Company Limited, 1862 Zishi Road, Hefei City, Anhui, China) in the national biorepository unit until they were used for further testing according to the evaluation protocol. Repeat testing using the same national HIV rapid testing algorithm was performed in a controlled laboratory environment by skilled laboratorians who were given refresher training before the testing using validated test kits. Also, a confirmatory assay using the Geenius™ HIV-1/2 Supplemental Assay (Bio-Rad Laboratories. Redmond, Washington, United States) was done for all the HIV positives in the field and at the NRL; all discordant HIV results between the field (HIV-positive) and NRL (HIV-negative); as well as all discordant results between the first and second tests done at NRL. The accuracy and reproducibility of the national HIV rapid testing algorithm performed at the NRL were confirmed by a higher platform Multiplex HIV-1/2 assay, Luminex^®^ MAGPIX^®^ (Luminex Corporation, Austin, Texas, United States). All the evaluation samples were further characterised using the Multiplex Bead Assay technology (Luminex Corporation, Austin, Texas, United States). Two laboratorians tested the samples (in duplicate) using the Multiplex Bead Assay platform, and discordant results (between the testers) were also retested (in triplicate) for concurrent HIV diagnosis and serotyping.

Survey participants’ inclusion and exclusion criteria: Clients aged 15 years – 64 years who sought HTS services at the selected health facilities and consented to participate in the evaluation were included, while clients aged less than 15 years and above 64 years, as well as those who did not consent to participate, were excluded from the evaluation.

### Quality assurance measures

#### Selection, training, and competency assessment of recruited personnel (quality assurance measure)

Proper selection and training of the field staff was one of the key measures to ensure the quality of the algorithm evaluation process. A hundred and three field staff were recruited for retrospective data abstraction, while eighteen experienced laboratory scientists were engaged as prospective phase data collectors and sample processing and shipment officers. Due to the high impact of the coronavirus disease 2019 pandemic at the time of the study, Nigeria imposed a national policy on physical meeting restrictions. This restriction resulted in an innovative cost-saving alternative model: a 5-day, remote virtual training, which did not compromise the quality of normal physical training. A total of 169 study personnel, including representatives from the government of Nigeria, were trained. At the NRL, three different groups of competent testers were recruited and given separate refresher trainings: seventeen (17) laboratory scientists who had earlier participated in the HIV/AIDS national impact survey of 2018 and had obtained technical experience on the functionality of the Geenius™ HIV-1/2 Supplemental Assay, five (5) laboratory professionals who were highly experienced in HIV serology rapid test, and two (2) laboratory professionals who were experienced with the multiplex assay. They were all required to achieve a 100% pass score in the competency assessment administered after the training to qualify as testers in the prospective phase of the survey at NRL.

#### Standardisation of laboratory processes in the field and at the National Reference Laboratory

To further assure the quality of laboratory activities, a laboratory operational manual was developed, which contained all the processes and procedures required from pre-examination to post-examination phases, from sample management to result transmission into the evaluation database. This document was printed and disseminated to laboratory staff involved in the prospective phase of the evaluation across the evaluation sites to ensure standardisation of processes towards a quality output.

#### Supervision

Field supervision ensured that best practices were followed and the study protocol was strictly adhered to at all project implementation phases. Two rounds of supervisory visits were conducted within 3 months during the field evaluation phase. All field supervisors comprising representatives from the implementing partners, National AIDS, Viral Hepatitis, and STIs Control Programme (NASCP), and National Agency for the Control of AIDS, were trained on field assessment before the visit. A checklist was developed and administered to assess compliance and adherence to the evaluation protocol. The site laboratory supervisor supervised on-site specimen collection across the selected sites for the prospective evaluation, per the protocol. The central technical supervisors (NASCP, National Agency for the Control of AIDS, United States CDC, and University of Maryland Baltimore) monitored the field sites to ensure that consent forms were duly completed, to assure quality and volume of specimens collected, and to confirm storage conditions. Specimen information was clearly and anonymously unlinked from patients’ identifiers. Issues observed in the first visit, such as irregularities in data entries, overshooting of data entry targets for negative samples, and deficiencies in field sample storage facilities, were addressed before the second visit. The United States CDC team and/or other vital stakeholders provided daily and scheduled technical and operational oversight on testing quality to ensure the laboratory-controlled environment was met and the associated evaluation protocol-oriented guidelines were followed throughout the testing at the NRL.

### Data collection

Test data for the retrospective and prospective evaluations were collected on mobile tablet devices using custom-built software (Online Supplementary Figure 1, Online Supplementary Figure 2, and Online Supplementary Figure 3). Each study participant was randomly assigned a unique identifier. All test data across the 12 states were transmitted and synchronised into a central server and dashboard using real-time monitoring and support systems. Only designated personnel had access to the data. All personnel involved in data abstraction signed a data confidentiality agreement.

### Data security (storage and ownership)

The collected data were stored on password-protected tablets provided only to the data entry clerks throughout the evaluation. The tablet information was backed up daily. The final data set was maintained and stored in a secure server at the University of Maryland Baltimore Central Office. All data and reports belonged to the Federal Ministry of Health.

### Data analysis

Results from the field testing using the National Rapid Testing Algorithm were compared with those from the laboratory testing at the NRL using the same algorithm. The positive predictive value and negative predictive value were calculated based on the reference testing results, using Geenius™ as the confirmatory test and the test performed at the NRL as the reference or standard (Table 3).

**TABLE 3:** A two-by-two table showing the level of agreement between test results and Geenius™ in Nigeria, July 2020 – September 2020.

Results of assay under evaluation	Geenius™ HIV-1/2 Supplementary Assay		
	Positive	Negative	Total
Positive	A	B	A + B
	True positives	False positives	
Negative	C	D	C + D
	False negatives	True negatives	

**Total**	**A + C**	**B+D**	

*Source:* Adapted From: Iriemenam NC, Mpamugo A, Ikpeazu A, et al. Evaluation of the Nigeria national HIV rapid testing algorithm. PLOS Glob Public Heal. 2022;2(11):e0001077. https://doi.org/10.1371/journal.pgph.0001077

### Dissemination

The evaluation outcome with a detailed report was shared with the government of Nigeria (NASCP, and National Agency for the Control of AIDS), PEPFAR, Global Fund, the National Laboratory Technical Working Group, and other critical in-country stakeholders, including multilateral organisations and implementing partners. Appropriate approvals were obtained, and consent was sought from the co-investigators and the government of Nigeria before study data were published. Study findings have been published in a peer-reviewed journal.^17^ The evaluation findings were further disseminated to stakeholders (including multilateral partners, bilateral partners, and implementing partners) at a forum made available by NASCP, to support a consensus with partners and stakeholders for a future policy decision. The evaluation complied with the PEPFAR Evaluation Standards of Practice requirements on final report accessibility.

## Lessons learnt

The findings of the main evaluation study, which have been published in another study,^17^ show higher positive predictive value and better concordance rates between the first and second tester when the indices at the NRL under controlled conditions were compared against the field evaluations. This evaluation method has some distinguishing features from similar studies across sub-Saharan Africa. First, while similar evaluations focused commonly on the performance characteristics of individual test kits of an algorithm,^11,18^ this evaluation method focused on assessing the quality of testing in the entire testing strategy of the current algorithm used in Nigeria. The benchmark for comparison was the algorithm performance at the NRL under controlled settings, such as using trained, competent personnel, controlled environmental temperature, quality-assured test kits, and adherence to testing protocols. The positive predictive values, algorithm agreement rates, and first/second test concordance rates across testing points were compared between the field and NRL.

This study method aligns with methods used in other similar studies where performance characteristics of test kits were compared across different testing points, prevalence settings, and regional, environmental, and personnel factors in algorithm selection.^19,20,21,22^ This evaluation followed the national HIV rapid testing algorithm strictly. The two-test serial rapid testing algorithm was used at the field level and in a controlled laboratory environment to compare performance, resolve discordances between the two settings, and discordance between the first- and second-line tests. The use of Geenius™ HIV-1/2 supplementary assay and HIV multiplex assay technology further confirmed the testing quality in both settings.^17^ Cameroon used a similar method to evaluate its two-test national HIV testing algorithm at the field level and in a controlled laboratory setting with quality management systems. The Cameroon National HIV testing algorithm also confirmed their field and laboratory testing using the Geenius™ HIV-1/2 supplementary assay.^23^

### Stakeholder engagement

Proper stakeholder engagement is a foundational stone in any project planning and implementation success.^24^ Before the commencement of the algorithm evaluation, key stakeholders were line-listed and consulted for their ownership and support toward the activity’s success. The United States CDC funded the project and provided technical and oversight functions; the government of Nigeria, through its agencies (NASCP and National Agency for the Control of AIDS), took ownership and provided leadership and control of project activities which gave the project unrestricted access to the government facilities through their chief medical directors and laboratory directors. Involving the PEPFAR implementing partners overseeing each site ensured adequate technical personnel, laboratory infrastructures, and unrestricted access to the selected sites across the states. Engaging the Nigerian Centers for Disease Control as an implementing partner in the evaluation provided unrestricted access to facilities and personnel at the NRL, as well as facilitating long-term storage of post-evaluation samples at the biorepository laboratory. The cumulative efforts of all stakeholders provided the synergy that produced a very successful evaluation.

### Constitution of the team of field supervisors

A well-composed field supervisory team that included government representatives from the national and state levels provided an acceptable and result-oriented supportive visit. A second supervisory visit to review the status of the recommendations on the non-conformities of the first visit was a quality improvement initiative that further impacted the quality of study processes and outcomes. Also, the regular visits of the United States CDC technical team to the NRL to ensure compliance with the expected controlled conditions for testing at the NRL gave rise to a high-quality outcome in laboratory results from the three groups of testers.

### Competency assessment: Criteria for selection of testers

Competent and qualified personnel are critical to the laboratory quality management system.^25^ Using competency assessment instead of pre- and post-tests in the selection process ensured that only competent personnel were recruited to conduct testing at NRL. This factor also significantly contributed to the quality of the evaluation process, such as a reduced number of repeat tests, minimal supervisor attention, and a high concordance rate between first and second tests.

### Innovative approaches to training and study design

Furthermore, this study innovatively employed virtual training and concurrent competency assessment methodology in addition to regular virtual and/or supportive physical supervision, where possible, due to coronavirus disease 2019 -associated restrictions, to ensure high-quality and optimal testing service delivery at the field and laboratory levels during the survey-implementation period. The retrospective component of the study enabled the team to review the paper-based National Daily HIV Testing Register for concurrence. In line with World Health Organization guidance on the country selection of suitable algorithm, this evaluation underscored the importance of periodic assessment of an entire algorithm under different settings and conditions beyond evaluating individual test kit performance.^9^

### Real-time data transmission and dashboard review

A custom-built data-capturing mobile app was installed on tablet devices and distributed to field staff for real-time data capture and transmission to a central server. A dashboard was created that provided vital analytics on the daily efforts in the field, and access was given to the critical project management team members to review, track, and coordinate field performance remotely on a daily basis across the 12 states. This strategy provided motivation, proper direction, and redirection of field activities across the states, which resulted in achieving a 96.5% (2895) sample collection within the set time frame of less than 3 months.^17^

### Limitations

The study methods did not include confirmatory testing for all the negative samples derived from the field and NRL using the Geenius™ HIV-1/2 Supplemental Assay. Due to cost constraints, only 10% of the negatives were subjected to confirmatory testing. Testing all negatives will increase the probability of finding more false negatives and accuracy in determining the true specificity of the algorithm under evaluation.

## Recommendations

This study methods provide a reference study design for periodic assessment of the national HIV rapid testing algorithm’s performance under different settings and conditions, beyond evaluating the performance of individual test kits. In addition, we recommend that all samples tested in the field and the reference laboratory be confirmed using a gold standard to identify all true positives and negatives.
